# Rhythm perception, production, and synchronization during the perinatal period

**DOI:** 10.3389/fpsyg.2014.01048

**Published:** 2014-09-18

**Authors:** Joëlle Provasi, David I. Anderson, Marianne Barbu-Roth

**Affiliations:** ^1^Laboratoire Cognition Humaine et Artificielle, Ecole Pratique des Hautes EtudesParis, France; ^2^Department of Kinesiology, San Francisco State UniversitySan Francisco, CA, USA; ^3^Institute of Human Development, University of California at BerkeleyBerkeley, CA, USA; ^4^Laboratoire Psychologie de la Perception, Université Paris Descartes – Centre National de la Recherche ScientifiqueParis, France

**Keywords:** synchrony, rhythm, sensorimotor synchronization, infants

## Abstract

Sensori-motor synchronization (SMS) is the coordination of rhythmic movement with an external rhythm. It plays a central role in motor, cognitive, and social behavior. SMS is commonly studied in adults and in children from four years of age onward. Prior to this age, the ability has rarely been investigated due to a lack of available methods. The present paper reviews what is known about SMS in young children, infants, newborns, and fetuses. The review highlights fetal and infant perception of rhythm and cross modal perception of rhythm, fetal, and infant production of rhythm and cross modal production of rhythm, and the contexts in which production of rhythm can be observed in infants. A primary question is whether infants, even newborns, can modify their spontaneous rhythmical motor behavior in response to external rhythmical stimulation. Spontaneous sucking, crying, and leg movements have been studied in the presence or absence of rhythmical auditory stimulation. Findings suggest that the interaction between movement and sound is present at birth and that SMS can be observed in special conditions and within a narrow range of tempi, particularly near the infant’s own spontaneous motor tempo. The discussion centers on the fundamental role of SMS in interaction and communication at the beginning of life.

## INTRODUCTION

Sensori-motor synchronization (SMS), the capacity to synchronize a rhythmic motor pattern with an externally perceived rhythm, is unique to species capable of vocal learning (Patel, 2006). SMS plays a crucial role in human communication because it enables one person to demonstrate to another that she perceives the other’s behavior and responds accordingly. A remarkable property of SMS is that two people can communicate by producing a synchronized rhythm with different behaviors. For example, a person could tap a finger while listening to another person singing or she could sing in cadence with that person. Virtually any combination of different behaviors could generate a means to communicate as long as the behavior produced a rhythmic pattern compatible with the perceived rhythm. Consequently, SMS has been studied in a large variety of contexts including, mapping rhythmic body movements like stepping, dancing, and finger tapping to the rhythm of a visual or auditory stimulus, and mapping more complex activities like playing music in synchrony with other players and speaking or singing in response to other vocalizations (Phillips-Silver et al., 2010). The only limit to the number of possible SMS combinations is the capacity to produce a rhythmic motoric pattern with some part of the body and the capacity to perceive an external rhythmic pattern via any of the perceptual systems – the combinations are almost infinite.

Not surprisingly, many studies have focused on understanding how these rhythmic couplings take place in various contexts. The majority of studies have examined the capacities of adults and children and most concern the coordination between motor behaviors and isochronous auditory patterns (for a review see Repp, 2005; Repp and Su, 2013). Research indicates that durations within the 100 ms range are more effectively processed when presented in auditory than in visual modalities and when presented through isochronous compared to isolated sequences (Schulze, 1989; Drake and Botte, 1993; Miller and McAuley, 2005). In short, all studies on the auditory-motor synchronization capacities of adults and children have found that SMS is more accurate when auditory tempi are close to the spontaneous motor tempi (also called the referent period) of each specific person (Jones, 1976; Jones et al., 1993). This period is typically estimated with the Spontaneous Motor Tempo task (SMT), in which the person is asked to tap in a comfortable and regular pace with the finger without an external pacing stimulus. The value of the SMT is around 600 ms in adults (Fraisse, 1974), around 700–800 ms in elderly adults (Vanneste et al., 2001), and around 400–500 ms in children 4 years of age and older (Drake et al., 2000; McAuley et al., 2006). According to Drake et al. (2000) the SMT is variable in children and becomes more regular as the child develops. Young children have a fast SMT with a limited synchronization range, but the SMS range widens and can adapt to slower tempi with increasing age.

In comparison to the extensive literature on adults and older children, few studies have examined younger children. This is in part due to the difficulty young children and infants have performing rhythmic actions when asked to do so in an experimental setting and the immature motor system’s ability to produce periodic patterns, especially when the child is in the process of learning the motor pattern. As a result, it is crucial that experiments on this young population take into account the young child’s capacity to spontaneously produce a rhythm with the effector under study. To maximize the chances of observing SMS in young children it is essential to choose a motor behavior that is easy enough for the child or infant to perform so that the opportunity exists to produce a rhythm matching the auditory or visual rhythm used in the study. For example, though infants from the age of 5 months are able to produce spontaneous rhythmic motions of their bodies in response to music (Zentner and Eerola, 2010), there is little evidence they can adjust the tempo of their body movement periodicities to match the tempo of the music (Eerola et al., 2006), even though faster auditory tempi generate faster movement tempi (Zentner and Eerola, 2010). In other words, increasing the temporal frequency of the auditory stimulus increases the number of body movements but infants are not able to perform SMS in this context.

To increase the likelihood of finding SMS in infants and young children, Provasi and Bobin-Bègue (2003) and Bobin-Bègue and Provasi (2008) tested a rhythmic motor behavior that is easier to adapt than a whole body movement – finger tapping in rhythm with an auditory tempo (animal noises played at different rhythms). They found that children between 18 months and 4 years of age were able to synchronize their movement with the external rhythm only if the temporal frequency of the auditory pattern was close to the spontaneous motor tempo of their finger tapping, i.e., around 400 ms. All ages were better at accelerating than decelerating and deceleration was associated with more variable synchronization than acceleration. Moreover, children around 1 year of age were more variable in their response than 4-year-olds. However, the variability between children increased with age as a result of a wider accessible range of tempi. Using finger taping in response to another type of auditory stimulation, children’s songs with tempi between 375 and 750 ms, van Noorden and De Bruyn (2009) confirmed that 3-year-old children have a SMT of 500 ms periodicity and these children can only synchronize in a very narrow range (around 500 ms) close to their SMT, similar to what was discovered by Provasi and Bobin-Bègue (2003).

Interestingly, social context seems to improve SMS. For example, Kirschner and Tomasello (2009) found that social context facilitates joint drumming synchronization in preschool children. Three age groups (2.5, 3.5, and 4.5 years) were asked to drum on a floor drum with a single hand at two different tempi (400 and 600 ms), with each task presented in three conditions: (1) a social condition with a human partner drumming, (2) an audio–visual condition with a drum machine, or (3) an acoustic condition with a drum sound coming from a speaker. Children of all ages synchronized their drumming with higher accuracy in the social condition than in the audio–visual and acoustic condition. Children as young as 2.5 years adjusted their drumming tempo to 600 ms, a beat which was outside the range of their spontaneous motor tempo, though they were less accurate than the older children. The authors concluded that drumming together with a partner motivates the child to perform SMS. After 4 years, when the variability of finger tapping decreases and the range of accessible tempi becomes wider and deceleration becomes possible, the SMS capacity progressively increases.

In summary, adults and children from 5 years of age onward are able to accurately synchronize their spontaneous motor tapping tempo to an external auditory rhythm if the rhythm is around their referent period and below one second. Younger children and infants are sensitive to rhythm because they increase their body movements when listening to a musical tempo that increases. However, they have difficulty synchronizing their movement to an external rhythm, even if the movement is as simple as finger tapping. To be successful in this type of task, children under 4 years of age must listen to a tempo that is at a minimum of 20% of their spontaneous motor tempo (500 ms in finger tapping; Bobin-Bègue and Provasi, 2005, 2008). These findings highlight three important points. First, an appropriate motor behavior and auditory tempo must be used to maximize the probability of seeing SMS in infants and young children. Second, the capacity of infants and young children to perceive and produce rhythms needs to be studied in order to determine the optimal contexts for testing SMS in early development. Third, despite limitations in SMS, infants, and young children are nevertheless capable of responding to rhythms by changing the quantity of their movements, especially by increasing the number of movements in response to accelerating tempi. This last result suggests that this quantitative motor response, even though not rhythmically adapted, could be a potential marker to follow as a precursor of SMS in early infancy. With this backdrop in mind, we will turn now to the primary focus of this paper; a review of the earliest capacities of humans to produce and perceive rhythms.

A systematic search for English-language articles on sensori-motor-synchronization in fetuses, neonates, and very young infants between 1888 and 2014 was performed. Half of the papers were recent studies (48% of the references after the year 2000) but pioneering studies were also included. Articles were identified from the following electronic databases: psychological database (PsycInfo) and medical databases (Pubmed, Medline). The search terms used were a combination of “tempo,” “rhythm,” “rhythmical synchronization,” “rhythmical production,” “sensori-motor-synchronization,” “neonates,” “fetuses,” “newborn.”

## RHYTHM PERCEPTION IN THE FETUS AND NEWBORN

The environment of the fetus offers multiple potential sources of rhythmic stimulation. For example, visual, vestibular, tactile, and somatosensory stimulation produced by the mother’s movements likely play an important role in producing the rhythmic patterns that surround the fetus (Lecanuet and Schaal, 2002). However, most studies on early rhythm perception concern the perception of rhythmic auditory stimulation. The bias toward studying auditory versus visual, vestibular, tactile, or somatosensory stimulation probably reflects two factors. First, it is much more difficult to systematically vary visual, vestibular, tactile, and somatosensory stimuli than auditory stimuli, particularly when the fetus is the target of exposure. Second, auditory stimulation, particularly the rhythmic stimulation included in speech, songs, and music are believed to play a pivotal role in the development of early infant communication via speech perception and production.

### PERCEPTION OF VESTIBULAR-TACTILE-SOMATOSENSORY (VTS) RHYTHMS

The morphology of the vestibular apparatus is mature by 14 weeks of age in the fetus (Lecanuet and Schaal, 1996). However, because each maternal movement stimulates the fetus’s vestibular system, Prechtl proposed the existence of a central mechanism that suppresses fetal vestibular responses (Prechtl, 1985). In essence, the same suppression hypothesis could easily be proposed for tactile or somatosensory responses given that maternal movement elicits a mixture of vestibular-tactile-somatosensory (VTS) stimulation. Despite Prechtl’s hypothesis, the fetus is sensitive to maternal movements as shown by fetal reactions to passive rhythmic displacements caused by their mothers’ movements. The limited studies in this area have used fetal heart rate (HR) as an index of responsiveness to displacement. Near term fetuses display different HR patterns when the mother is walking (rhythmic patterns) versus resting while sitting or resting in a reclining position (Cito et al., 2005).

The above results suggest that fetuses are responsive to maternal movements but they do not show that they can perceive different rhythms associated with maternal displacements. Lecanuet and Jacquet (2002) have tested this hypothesis. They have shown that by the end of gestation, fetuses display HR changes in response to passive, rhythmic maternal displacement, either in the antero-posterior direction in a rocking chair or in a lateral direction in a swaying garden glider. In this experiment, mothers were moved for 5 or 26 s with a rocking tempo of 2.6 s for a complete cycle and a swaying tempo of 2 s for a complete cycle. The average HR obtained during 5 or 26 s of rocking was significantly higher than that obtained in the non-stimulated control group. The rocking HR acceleration was similar to that obtained with a rhythmic pressure on the maternal abdomen at a rate of 1 press per second (Porton-Deterne and Le Du, 1997). Moreover, the average HR obtained during 26 s of rocking was significantly higher than the average HR observed during 26 or 5 s of swaying.

If the fetus perceives the difference in the rhythm of maternal rocking versus swaying, would it also perceive the different rhythms of maternal walking? This ability has never been demonstrated even though maternal walking is the major type of passive movement experienced by the fetus. It would be interesting to investigate whether fetuses are already tuned to the specific pattern and rhythm of their own mother walking or if they can distinguish between walking patterns generated by different humans or animals. We believe this question has especially important implications for the medical care of premature neonates, who can spend several weeks outside the maternal environment prior to the date they would have been born had they been carried to term. While the consequences of this deprivation on physiological parameters have been studied extensively, fewer studies have focused on the consequences for sensori-motor development. Although the effect of the infant not being passively transported by the mother is difficult to investigate, several studies have shown that premature infants benefit from rhythmical rocking (Barnard and Bee, 1983; White-Traut and Nelson, 1988; Clark et al., 1989; Groswasser et al., 1995). These studies of rhythmical stimulation (direct rocking, rocking mattress, sinusoidal oscillation, rocking associated with heartbeat stimulation every hour or depending on infant self-activation) in premature infants have consistently shown that stimulated infants have better weight gain, less obstructive breathing events, fewer abnormal reflexes, and better orienting responses than non-stimulated infants.

Interestingly, rhythmical stimulation also has a major effect on rhythmical respiratory rate. Sammon and Darnal (1994) recorded respiratory abdominal movements in 18 premature infants who were manually rocked at a rate varying from one rock every second to one rock every 1.5 s. The authors found that 15 of 18 premature infants increased their respiratory movements to two breaths per rocker cycle, with the best result occurring in response to the slowest rocking tempo. Premature infants older than 35 weeks gestational age exhibited greater synchronization to rocking than premature infants born before 35 weeks gestational age. The authors concluded that the rocking stimulation entrains the respiratory pattern generator. In a more recent study, Zimmerman and Barlow (2012) compared the breathing rhythm of preterm infants who were exposed to a series of vestibular stimuli that were counterbalanced across rate and acceleration for 15 min sessions three times per day for 10 days to a control group that did not receive any vestibular stimulation. The vestibular stimuli were linear horizontal displacements of the whole body at rates and accelerations within the range of those typically produced by preterm breathing. The results showed that infants who received vestibular stimulation increased their respiratory rate significantly compared to infants who received no stimulation. Moreover, the vestibularly stimulated infants were able to adapt their breathing to the rate and acceleration of the vestibular stimuli, with the highest vestibular acceleration inducing the highest number of breaths per minute. As this type of adaptive breathing response is vital for adjusting to various task demands, such as feeding and early vocalizations, this type of vestibular stimulation has important implications for the preterm infant, beyond the immediate benefit of potentially gaining control of the breathing mechanism more rapidly.

The above-mentioned studies suggest that the fetus can perceive VTS stimulation and can differentiate VTS rhythmic patterns. Moreover, this type of stimulation appears crucial for the development of the fetus. Not surprisingly, newborns are especially sensitive to this type of stimulation and caregivers use it in all cultures to calm infants. This is the case when infants are passively transported by the mother in different positions and when the infant is rocked in a stroller or bed (we elaborate later on the capacity of older infants to recognize vestibular rhythms).

### PERCEPTION OF AUDITORY RHYTHMS

In contrast to the perception of passive VTS rhythmic movements, the perception of rhythmic auditory stimuli has been studied extensively in fetuses and newborns over the last 30 years. During fetal life, rhythmic auditory stimuli originate from two sources: (1) inside the womb from movements of the fetus or the mother, and (2) from the external environment. What is known about exposure of the fetus to rhythmic auditory patterns originating from the womb? In the first recordings of the sounds inside the mother’s womb, researchers concluded that the fetus was exposed to a continuous background of loud noises coming from the maternal cardio-vascular system (Murooka et al., 1976; Busnel, 1979). However, the more sophisticated hydrophones and narrow-band analyses used in recent investigations indicate that the womb is a relatively quiet place (Lecanuet and Schaal, 2002). In the absence of external auditory stimuli, the womb’s background noise is constituted of biological rhythmic stimulation that arises from maternal respiratory activity as well as maternal and fetal cardio-vascular rhythms.

Most studies of fetal perception of auditory rhythms have focused on the perception of maternal heartbeats by the fetus. Maternal heartbeats are approximately 25 dB (Querleu et al., 1981, 1988) and consist of low-pitched pulsations at frequencies between 200 and 800 Hz (Walker et al., 1971; Murooka et al., 1976; Querleu et al., 1981; DeCasper and Prescott, 2009). Maternal heartbeats are probably the most salient rhythmic auditory stimuli in fetal life since the fetus is exposed to a stream of more than 5 × 10^6^ rhythmic maternal heartbeats between 32-weeks gestational age and birth (Rayson et al., 1997; Mehl et al., 2007). Magnetoencephalographic studies have provided consistent evidence that the fetus can respond to external stimuli and to the sound of the mother’s heartbeat (Porcaro et al., 2006). Moreover, as the maternal cardiac rhythm changes according to the mother’s activity and stress level, the fetus has the opportunity to experience different patterns of rhythmicity. Can the fetus perceive these changes? Fetuses of mothers with high maternal anxiety show significant HR increases during maternal cognitive stress compared to fetuses of mothers with low maternal anxiety who show non-significant HR modification under the same condition (Monk et al., 2000). However, it is not known whether this phenomenon is perceptual in origin or whether it emanates from a physiological process. Interestingly, like rocking, exposure to the sound of the maternal heartbeat is often used to calm newborns (Smith and Steinschneider, 1975; Rosner and Doherty, 1979). The intensity of the behavioral response to stress decreases considerably in neonates who are exposed to the sound of the maternal heartbeat compared with those who hear the beat of a drum (Kurihara et al., 1996). The sound of the heartbeat is also used as positive reinforcement in newborn operant conditioning (DeCasper and Sigafoos, 1983).

While maternal heartbeats are important for the fetus, they have failed to generate as much scientific interest as maternal speech and music. This is probably due to the pivotal role accorded to maternal speech/voice and music in the development of the infant’s speech and communication. However, many components of maternal speech/voice and music could play a role in the future development of the infant’s vocal communication. We will briefly review some of them to highlight the role of rhythm in the perception of the voice, speech, song, and music in fetuses and neonates.

### VOICE AND RHYTHM PERCEPTION

The maternal voice and external speech emitted close to the mother are easily perceptible by the fetus if they are over 60 dB (Lecanuet et al., 1995; Lecanuet and Schaal, 2002). Although speech is significantly attenuated in the womb, its prosodic (e.g., rhythm and pitch) characteristics are preserved (Busnel, 1979; Spence and Decasper, 1987; Querleu et al., 1988), imparting rhythm and pitch contours (Moon et al., 2013). Between 32-weeks gestational age and birth, the fetus is exposed to a stream of about 6.7 × 10^5^ words occurring in normal conversational utterances (Rayson et al., 1997; Mehl et al., 2007). In this auditory environment, what sounds can the fetus perceive? What kind of information can the fetus process? Fetuses respond to their mothers’ voices as early as 32–34 weeks (Kisilevsky and Hains, 2011) and near term fetuses can detect and respond to the maternal voice (Voegtline et al., 2013). They can also discriminate their mother’s voice from the voice of a female stranger (Kisilevsky et al., 2003), suggesting that the fetus has learned, memorized, and recognized some property of the mother’s voice. This learning is probably based on the prosodic characteristics of the voice as these speech features are the ones best preserved in utero. Six months later, infants discriminate voicing on the basis of temporal envelope cues (Bertoncini et al., 2011). More specifically, newborns recognize and prefer familiar speech sequences that their mothers spoke aloud during the last week of their pregnancy compared to new speech sequences even if the familiar speech sequence was spoken by a non-familiar voice (DeCasper and Spence, 1986). The fetus not only learns the specific features of the mother’s voice but also cues such as the syllabic beat, the voice-onset-time of consonants, the harmonic structure of sustained vowel sounds, and/or the temporal order of these sounds (DeCasper and Spence, 1986).

### SPEECH AND RHYTHM PERCEPTION

The temporal variations in speech, i.e., the rhythmic properties of language, play a major role in the development of language discrimination in infancy (Bertoncini et al., 1995; Nazzi et al., 1998; Ramus et al., 2000). At birth, newborns are not only able to discriminate their native language from a foreign language (Mehler et al., 1988; Moon et al., 1993) but can also discriminate two different foreign languages on the basis of prosodic information (Nazzi et al., 1998). Prosodic features such as melody, intensity, and rhythm are essential for language acquisition (Vihman, 1996). Newborns already extract prosodic, more specifically rhythmic, properties of sentences, and sort sentences into classes based on rhythmic, timing properties (Ramus et al., 1999; Byers-Heinlein et al., 2010; May and Byers-Heinlein, 2011; Moon et al., 2013). Womb experiences affect vowel perception earlier than consonant perception because vowels are louder, longer in duration, and carry salient prosodic information such as melody, rhythm, and stress (Moon et al., 2013).

Human newborns discriminate languages from different rhythmic classes, fail to discriminate languages from the same rhythmic class, and fail to discriminate languages when the utterances are played backwards. Infants are sensitive to rhythmic language patterns and this sensitivity is a key factor in launching the process of language acquisition (Petitto et al., 2001). The prosody allows infants to detect emotional changes in the voice (Cheng et al., 2012) and to recognize different emotional expressions in the voice (Schneider and Fischer, 2011). Emotional prosody is shared in communication but also in music and in gestures (Huotilainen, 2013): “From this point of view, prosody may well be the most multisensory part of language and … we may call prosody ‘our first language’ ” (p. 103).

### SONG AND RHYTHM PERCEPTION

Newborns recognize and prefer melodic sequences that their mothers sang repeatedly during the last weeks of pregnancy (Cooper and Aslin, 1989). Moreover, near term fetuses are able to discriminate between a familiar children’s rhyme recited aloud by their mother during the last 4 weeks of pregnancy (fetal HRs decrease) and a novel rhyme that had not been heard before (no change in fetal HR; Decasper et al., 1994). In the DeCasper et al. study, the two rhymes were tape recorded by a female stranger. Five target rhymes, two in French and three in English, were used during a five-repetition familiarization phase or served as control rhymes. Consequently, the familiarity effect of the repeated rhyme was due to the acoustic characteristics that were not specific to the maternal voice, a specific language (French or English), or to the unique acoustic characteristic of any one passage. The authors concluded that the characteristics allowing the fetus to discriminate rhyme might include intonation contours, and/or the pattern of syllabic beats, the harmonic structure of sustained vowel sounds, and/or the temporal order of these sounds. Thus, rhythm seems to again play a role here. In further support of this idea, it is interesting to note that near-term fetuses are not able to discriminate two piano melodies presenting the same tempo and the same rhythm, even if the melodies have opposite contours (Granier-Deferre et al., 2011). Similarly, the same fetuses are not able to discriminate two sentences, one in Icelandic and another its chimera, with both having exactly the same sequence of rapid and slow temporal amplitude variations over time, but not having the same phonological information.

### MUSIC AND RHYTHM PERCEPTION

James et al. (2002) have reported the existence of fetal learning during repeated exposure to music. In their study, fetuses were exposed to the same piece of music for 4 h (3-min recording 80 times). Compared to infants in a control group who were not exposed to music, infants exposed to music started to show more reaction to this music during the fourth hour of exposure (exhibiting more state transitions and higher fetal HR variation). After birth, all the newborns were exposed to the same music on postnatal days 3–5. Prenatally exposed newborns manifested more state transitions and spent more time in an awake state when exposed to the same music stimulus. The authors concluded that the fetus can learn to recognize music. In another study, near term fetuses were shown to discriminate between two low-pitched musical notes (Lecanuet et al., 2000) and to recognize a 600 ms inter-onset interval of an isochronous sequence of musical notes from a 10% faster or slower isochronous sequence (Lecanuet, Jacquet and Bontemps, cited in Granier-Deferre et al., 2011). Kisilevsky et al. (2004) also observed an overall HR deceleration in near term fetuses when they listened to a 5 min Brahms’ lullaby, regardless of the sound levels tested (95, 100, 105, or 110 dB). However, the fetal HR was significantly different if the music was presented at a normal tempo (69 beats per minute) compared to a tempo played a third faster (118 beats per minute). The authors concluded that near-term fetuses detect the tempo change in music and that tempo is a salient stimulus for near-term fetuses, suggesting a continuum in pre- and post-natal music perception.

At birth, newborn infants detect the beat in music (Winkler et al., 2009). Using auditory event related brain potentials (ERP), the authors showed that newborn infants detect a violation of the beat in a rhythmic sequence of sounds. In this experiment, the beat was extracted from a sequence comprising four different variants of the same rhythmic structure. These results suggest that the capacity to detect the beat in rhythmic sequences of sounds is already functional at birth and that newborns are able to detect regular features in the acoustic environment despite the four different variants of the same rhythmic structure. Infants possess the spectral and temporal processing prerequisites for music perception. From 2 months of age, infants perceive variations of rhythm (Demany et al., 1977), detect rhythmic pattern differences (Hannon and Trehub, 2005), and detect accelerations of 15% when the reference tempo is 600 ms (Baruch and Drake, 1997).

In summary, voice, speech, and music all contain different rhythmic components and these components are recognized by the fetus and the newborn. As these rhythmic components are the most predominant auditory signals in the womb, it is reasonable to hypothesize that they play a crucial role in the perception and learning of voice, speech, and music in the young infant. These results highlight the importance of using the appropriate characteristics of rhythm to allow the infant to detect language and music and learn the primitives of communication as early as possible. However, communication can start without using the complexity of signals included in voice, speech, and language because a communicative message can be transmitted with the simple synchronization between a motor behavior and a rhythmic auditory signal. Before turning to these capacities in neonates, we will focus briefly on how multimodal presentations of rhythm improve rhythm perception and what rhythms neonates are able to produce.

### CROSS-MODAL PERCEPTION

We know from the literature that the presentation of a rhythmic pattern in two modalities compared to one increases the capacity of the infant to recognize and respond in synchrony with the pattern (Bahrick and Lickliter, 2000; Bahrick et al., 2002). In adults, combining the audio and VTS modalities leads to the greatest increase in rhythmic perception, while visuo-tactile and audio–visual combinations are less effective (Fujisaki and Nishida, 2009). Is the link between VTS and auditory rhythm perception present at birth or even before birth? Lecanuet and Schaal (1996) proposed that the presentation of the same rhythm in auditory and somatosensory modalities improves fetal processing of the co-occurring cross-modal sensory inputs. Most of the rhythmic and passive movements experienced by the fetus, which are generated by the mother, are also experienced in the auditory modality. For example, a fetus that perceives its mother’s walking can often simultaneously perceive the auditory rhythm generated by the mother’s feet walking on a rigid surface. Kisilevsky and Muir (1991) recorded the fetal HR and movements of fetuses who received vibro-acoustic stimulation. They found that fetal responses increased when the rhythmic stimulation was presented in both auditory and vibratory modalities compared to only one sensory modality. Interestingly, studies using vibro-acoustic stimuli report much higher response rates than those using only sound or only mechanical vibration (Kisilevsky et al., 1992).

Since visual information is only vaguely experienced prior to birth, it is likely that cross-modal temporal synchrony is primarily experienced with the coupling of somatosensory (or VTS) and auditory signals during fetal life. This coupling continues after birth. Phillips-Silver and Trainor (2005) have shown that 7-month-old infants who are bounced in synchrony with a marching rhythm will prefer to listen to an auditory version of the marching rhythm pattern, which included intensity accents every second beat, compared to another marching rhythm with accents on every third beat. Another group of 7-month-old infants who were bounced in synchrony with a waltz rhythm (accent every third beat) preferred listening to an auditory version of the rhythm pattern that included intensity accents every third beat compared to one that had accents every two beats. The authors concluded that passive movements generating vestibular, somatosensory, and tactile stimulation influence the perception of auditory rhythms in human infants (Phillips-Silver and Trainor, 2005, 2007). More generally, vestibular information may contribute to perception of beat-based information (Todd, 2001; Todd and Lee, 2007).

## RHYTHM PRODUCTION IN FETUSES AND NEWBORNS

Producing rhythms is a tool used to communicate with others. Like adults, fetuses, and neonates are able to produce different rhythmic patterns. However, not all of these produced rhythms are immediately perceptible in the external environment of the neonate and fetus. Some of them are internally expressed and not easily accessible, especially during fetal life. These rhythms include cardiac pulsations, respiratory movements, hiccups, sucking, nasal breathing for olfaction, and swallowing for gustation. Although these rhythmic activities are not the best candidates for communication with others, they are all recordable and provide potentially good markers to index fetal and neonatal reaction to and synchronization with an external rhythmic stimulus. As described previously, fetal HR is still one of the most common markers used to follow the reactions of a fetus to external stimuli. The situation is different after birth. In addition to the activities mentioned already, the newborn is able to cry and move different body parts to express and communicate internal states and needs. Crying is the more powerful tool to alert the caregiver because crying not only permits distal communication but also can convey variable communicative signals via changes in crying melody, contour, and rhythm.

Since newborn crying is considered a precursor to language and music production, it is not surprising that it has been largely studied in comparison to newborn body communication. Interestingly, there are also more studies on vocal communication than on body movements in the literature on adult communication. However, as body movements are known to play a major role in adult communication, they should presumably play a major role in early interactions, especially in the context of the close peri-personal spaces shared by the infant and the mother in the first months after birth. For example, facial movements, such as eye and lip movements, are perceived by the mother and the infant at birth (see this special issue), but movements of the fingers, hands, legs, and head likely also convey crucial information within the dyad. We will now review what kinds of rhythmic patterns can be produced by fetuses and newborns and discuss their potential importance for sensorimotor synchronization and early interactions.

### CARDIAC PULSATIONS

The first cardiac pulsations of the fetus start as early as 3 or 4 weeks of gestation (Eichorn, 1970; de Vries et al., 1982). These pulsations are obviously rhythmic and never stop but lack variability initially. The extensive literature on fetal HR variability reveals that it appears at approximately 22–25 weeks of gestational age (Dreyfus-Brisac and Minkowski, 1968). By 28 weeks of gestational age, HR variability becomes increasingly correlated to what fetuses perceive around them and what movements they produce (Watanabe et al., 1973). High-intensity stimuli are associated with HR acceleration and low-intensity stimuli are associated with HR deceleration in the fetus (Graham et al., 1983). However, a developmental change occurs after birth with the emergence of a significant heterogeneity in the neonatal HR response (Graham and Jackson, 1969; Clarkson and Berg, 1983; Ockleford et al., 1988; Groome et al., 2000) that may reflect individual differences in the level of nervous system maturation at the time of postnatal testing (Ockleford et al., 1988). For example, the same stimulus that elicits a sustained HR deceleration in fetuses during quiet sleep might elicit a prolonged HR deceleration in half of the same infants tested at 2 weeks of age, with the other half responding with HR acceleration followed by HR deceleration (Groome et al., 2000).

### BREATHING MOVEMENTS

Ahlfeld (1888) was the first to observe breathing movements in the fetus. They emerge at 10 weeks of gestation and are spontaneously generated (de Vries et al., 1982; Lüchinger et al., 2008). However, these breathing movements are only observed episodically before 20 weeks of gestation and become regular around 32 weeks of gestation (Pillai and James, 1990; Roodenburg et al., 1991). The frequency of the movements increases, especially between 26 and 30 weeks. These regular movements involve rhythmic contractions of the diaphragm (Blackburn, 2012) and they last no longer than a second (Patrick et al., 1978). The variability of fetal breathing movements depends on environmental factors linked to the mother’s state. For example, fetal breathing movements could increase or decrease as a function of pathophysiological conditions of the mother, such as diabetes, hypertension, hypercapnia, smoking, and alcohol consumption (Florido et al., 2008; Einspieler and Prechtl, 2011). As described earlier in the section on the perception on VTS rhythms, in preterm infants, these breathing movements can be modified by external factors such as vestibular stimulation. In newborns, the rate, depth, and regularity of breathing are closely related to their behavioral state (Read and Henderson-Smart, 1984).

### HICCUPS

Hiccups are a typical fetal behavior consisting of phasic contractions of the diaphragm, usually repeated at regular intervals over several minutes. They have been observed as early as 8 weeks of gestational age (de Vries et al., 1982; de Elejalde and Elejalde, 1985) and occur frequently during the first trimester of gestation (Reinold, 1973). Hiccups are more common before 24 weeks of gestation than later (Pillai and James, 1990). Hiccups occur episodically, in regular succession, at an interval of 2–3 s, during an episode of a maximum of 10 min (van Woerden et al., 1988). Hiccups occur mainly as a succession of active cycles (Pillai and James, 1990) and are less present in at-risk fetuses (Einspieler and Prechtl, 2011).

### SUCKING

Sucking emerges at around 10 weeks of gestational age (de Vries et al., 1982). By 15 weeks, finger sucking is observed (Blackburn, 2012) and regular rhythmic jaw movements are reported as early as 20 weeks of gestation (Roodenburg et al., 1991; Einspieler and Prechtl, 2011). Sucking and swallowing activities increase as pregnancy progresses. At the end of gestation, regular mouthing movements are considered to be the same movements as “spontaneous sucking movements” and “non-nutritive sucking,” terms commonly used for neonates (van Woerden et al., 1988). Rhythmical and spontaneous movements of the mouth occur in clusters at a rate of approximately 1–2 per second. They last for approximately 2–4 s and are composed of 3–6 movements (Wolff, 1968; Dreyfus-Brisac, 1970). Rhythmical mouthing movements occur mostly during quiet sleep (1F state, Nijhuis et al., 1982). Interestingly, rhythmical mouthing movements and fetal HR patterns share the same rhythmicity from 38 weeks gestation (Otera et al., 2013); rhythmical mouthing movements are accompanied by concomitant and transient increases in FHR and FHR returns to baseline during the mouthing movement pause, before increasing again at the next sucking cluster. These concomitant behaviors led Otera et al. (2013) to propose a common Central Nervous System Center controlling both rhythmical mouthing movements and FHR responses.

After birth, neonates continue to suck either as a non-nutritive activity or in order to take food from the mother’s breast or a bottle. Both activities are highly rhythmic and can be modified in response to environmental factors, usually accelerating to improve the process of nutrition intake or in response to other rhythmic stimulations (see section below). However, the pause duration between bursts of sucks in non-nutritive newborn sucking can decrease (accelerating the sucking frequency), but not increase (Pouthas et al., 1996). The spontaneous sucking period of non-nutritive sucking is around 450 ms (Bobin-Bègue et al., 2006). During maternal breast feeding, the sucking pauses occurring at the end of feeding are believed to be important in the process of infant–mother interactions; in general, mothers move their infants to re-stimulate their sucking behavior and the alternation of sucking bursts and pauses generate “talking turns” between the mother and infant (Lecanuet, 2007). This precocious interaction is pivotal in shaping the social dialog between the mother and her infant.

### CRYING

Crying is a common newborn activity. It consists of a rhythmical series of sounds that requires precise coordination between the face musculature, the airway, and respiration (Hopkins and von Wulfften Palthe, 1987). Thus, crying is composed of a vocal and a non-vocal component. Wolff’s (1969) classical description of the infant cry corresponds to a rhythmic repetition consisting of an expiratory sound lasting 0.6–1.3 s, a brief pause of approximately 0.2 s, and an inspiratory sound of approximately 0.1–0.2 s followed by another pause of approximately 0.2 s before the next expiratory sound. In total, the crying frequency period is between 1100 and 2400 ms according to Brennan and Kirkland (1982).

The fundamental function of postnatal crying is vocalization. While it is not possible to record crying in the fetus, as the vocal component occurs only with the transition to extra-uterine life, Hopkins (2000) suggests that the non-vocal components of crying are developed well before birth. More recently, Gingras et al. (2005) observed from fetal ultrasounds that the fetus is capable of complex motor behaviors similar to crying. The components described by Gingras et al. (2005) are comparable to those described by Wolff (1969), with the vocalization component occurring during the expiratory phases. Fetal crying behavior, or state 5F, has been seen only after vibro-acoustic stimulation, suggesting that the “fetal cry” is elicited only when the fetus is disturbed (Gingras et al., 2005).

Crying is considered to be the first vocal precursor to language, with crying melodies becoming more and more complex during the first months of life and infants becoming increasingly able to modify their crying rhythm by varying the arc duration and the number of melody arcs within one cry (Wermke and Mende, 2009). Wolff and Simmons (1967) observed specific communicative sequences in the temporal crying pattern of newborns and noted differences between the basic cry and the pain cry. Specifically, when newborns cry in response to pain, their vocal expiration is five or six times longer than the expiratory vocalization of a basic cry and it is followed by a long silence during expiration that may last up to seven seconds. When a tape recording of a pain cry is played to mothers, they all respond with distress behaviors. However, if the long silence is removed in the tape, mothers uniformly respond with less distress. Wolff’s results suggest that the temporal arrangement of vocalizations has an important communication function even in the newborn period.

Recently, Mampe et al. (2009) reported that newborns can also vary their crying melodies because these melodies are already shaped according to the infant’s native language. In analyzing and comparing the crying melody patterns of 30 French and 30 German newborns, these authors found that French and German crying melodies are related to the intonation patterns observed in each language. This result highlights a very precocious capacity of newborns to respond to their native language. The authors concluded that when newborns produce sound they are already able to reproduce some of the prosodic properties of the specific language that they were exposed to prenatally. It is also interesting to note that precocious adaptation has been observed for the rhythmic characteristics of newborn crying as newborns can change the rhythmicity of their cries in response to external rhythmic stimuli (Provasi and Barbu-Roth, 2009). Finally, as infants grow older, they become more and more efficient at voluntarily controlling these rhythmic communicative sequences during interactive processes like mother–child musical- and language-learning activities (Wermke and Mende, 2009).

### ARM AND LEG MOVEMENTS

Movements of the arms and legs can be observed as early as 12 weeks of gestation, but are not well defined at this stage (de Vries et al., 1982). Between 13 and 16 weeks, the number of flexion and extension movements of the limbs increases and a more mature leg movement pattern emerges, consisting of rhythmical steps occurring mostly in bursts associated with a whole-body rotation of the fetus (Reinold, 1979). At this stage, a step cycle consists of a flexion of the leg up toward the body of the fetus followed by a short pause and then an extension of the leg downward. These rhythmical pumping movements of the limbs have been extensively studied in rat fetuses and have been shown to result from a coordinated pumping action of the limbs onto the elastic cavity of the uterus, in order for the fetus to move in the cavity (Brumley and Robinson, 2013). Interestingly, arm movements are sometimes observed in coordination with leg stepping movements in humans, as if the fetus is swimming or crawling in the mother’s womb (Kurjak and Chervenak, 2011). However, most studies of fetal movements attribute arm movements only to the function of reaching and leg movements to the function of displacement, despite the difficulty associated with ascertaining the goal directedness of fetal movements. Consequently, it is possible that some leg movements are dedicated to reaching and some arm movements are dedicated to stepping.

With regard to the above-mentioned hypothesis, it is interesting to note that Kuno et al. (2001) described fetal arm movements as usually occurring in bursts with intervals of less than 1 s and fast rhythmical movements of about three to four per second. These rhythmical patterns are closer to the description of a stepping activity than a reaching activity. Finally, de Vries et al. (1985) reported that fetal arm movements increase until 19 weeks of gestation, while leg movements decrease after 15 weeks. It would be interesting to know if this differential development is due to the biomechanical constraint of the legs having insufficient space to move compared to the arms and/or to a progressive dissociation of the function of the arms and legs. We do know from Kuno’s 3D ultrasound monitoring of fetal movements that arm and leg movements are the most active fetal behaviors during the second trimester of pregnancy (Kuno et al., 2001). These rhythmical limb movements continue after birth, with the newborn stepping in an upright position when his feet contact a rigid surface or kicking in a supine position. In fact, there is a striking similarity between fetal movements and postnatal motor patterns, suggesting the existence of a close continuity from fetal to neonatal behavior (Kurjak et al., 2004).

Newborn and infant rhythmical stereotypies have been studied extensively in the last thirty years, with Thelen (1979) identifying 47 instances of stereotypies. However, a growing number of studies are now showing that many neonatal leg and arm movements can be adapted in response to a variety of factors. Arousal is one factor as an increase in excitability increases the frequency of movements in the newborn. In addition to internal factors, newborns were recently shown to be capable of adapting the frequency of their leg stepping movements to visual stimuli and tactile stimuli (Barbu-Roth et al., 2009, 2013). In summary, human fetuses and newborns are able to produce rhythmical arm and leg movements and to modulate these movements in response to external stimulation.

### OLFACTION AND GUSTATION

Anatomical evidence shows that from the third trimester of gestation all chemosensory systems of the nose appear capable of processing sensory stimuli (Schaal et al., 1995; Lecanuet and Schaal, 2002). Although a smell is not a rhythmic stimulation, the breath, which is necessary and essential to perceive odors, is a rhythmic activity. Moreover, although a flavor is not a rhythmic stimulation, swallowing, which is also essential to perceive tastes, is a rhythmic activity.

In conclusion, fetuses and newborns are able to produce many rhythmic activities, most of them generated automatically at a subcortical level, but modifiable by internal and external stimulations. The primary question is whether *the rhythm* of these activities is modifiable in response to an externally presented rhythm, indexed either by a change in the frequency of the rhythmic activity in response to a specific external tempo or by a real SMS with this tempo. Studies have rarely addressed this question in the fetus, except the FHR studies described earlier, and few studies have been conducted with newborns. Before describing the newborn studies we will briefly outline why rhythm is a key factor in communication and why investigating the capacity of neonates to perceive, produce and synchronize their rhythms with other rhythms is essential to our understanding of the primitive capacities that underlie communication.

## ROLE OF RHYTHM IN EARLY INTERACTION

We have already established that fetuses and newborns are able to perceive and produce rhythms. These capacities do not imply that they are able to synchronize their rhythmical activity with external rhythmic information and use it for communication. While it is quite complicated to study synchronization in the fetus, it is less difficult to examine the impact of rhythm in newborns. Curiously, very few studies (see below) have investigated whether the presentation of a rhythmic stimulus induces a rhythmic response in the newborn. In contrast, most studies of early interactions have focused on either one) the capacity of the infant to interact in a rhythmical dialog with an adult caregiver or two) whether rhythmical information in the adult voice or music enhances neonatal attention and stimulates interactions. The former question relates to the concept of synchrony because it focuses on the temporal organization of the bond between the parent and infant and has been observed in coordinated gestures and vocalizations (Trevarthen, 1977, 1993; Trevarthen and Aitken, 2001; Feldman and Eidelman, 2007; Feldman, 2009). Based on comprehensive frame by frame analyses of mother–infant interactions in studies that date back to the 1960s, Trevarthen (1979) have concluded that infant’s movement patterns are very responsive to the time structure of their mother’s movements. This temporal organization of interactions is believed to play an elemental role in early social communication in humans (Trevarthen, 1979). For example, as early as 1974, Condon and Sander reported that newborns were able to synchronize their leg movements with the articulated structure of adult speech. Moreover, they found that newborns moved faster when an adult spoke faster, but did not move more slowly when an adult spoke more slowly (Condon and Sander, 1974). Later, but before 6 months, when the mother slows down the infant at first seems perplexed but then is able to adjust to the slowed speech (Tronick et al., 1979).

With regard to the capacity for rhythmical information to enhance neonatal attention and stimulate interactions, many studies have highlighted that the prosody in human speech as well as the beat in music can increase attention and interaction in infants. In fact, adults spontaneously modify the prosodic–melodic contour of their voice to interact with their infant (Gratier, 2007). Papoušek and Papoušek (1991) performed comparative acoustic analyses of German, Chinese, and American parental melodies during pre-linguistic parent–infant interactions. They found that the melodic contours of maternal speech are exaggerated in each culture, particularly on the most common types of melodic contours – rising, falling, and bell-shaped (Papoušek et al., 1991). Mothers use contrasting contours as a function of the infant’s arousal. Similarly, as early as 4 weeks of age, infants prefer this type of infant-directed (ID) speech than adult-directed (AD) speech (Cooper and Aslin, 1989; Pegg et al., 1992; Werker et al., 1994; Kaplan et al., 1995). The distinctive features of ID speech are not only concentrated on its melodic contour, but also include heightened pitch, exaggerated pitch contours, and a slower tempo compared to AD speech (Stern et al., 1982; Papoušek et al., 1990). ID speech is widely used across cultures (Ferguson, 1964; Grieser and Kuhl, 1988; Papoušek et al., 1991). Interestingly, adults in all cultures spontaneously vary rhythmic features (different tempi and different pitch contours) to talk to neonates and heighten their attention and responsiveness (e.g., Fernald and Kuhl, 1987; Singh et al., 2002).

Analyses of vocal exchanges between infants and parents reveal that infants do not vocalize randomly (Gratier, 2003) but take turns during the conversation (Snow and Hoefnagel-Höhle, 1977; Trevarthen, 1977; Bateson, 1979; Trevarthen, 1993; Henning et al., 2005). Gratier and Devouche (2011) found that 3-month-old infants actually imitate and repeat the prosodic contours of the parent during vocal interactions. They argue that preverbal infant vocalization is a function of the prosodic contour of the parent’s vocalizations (Gratier and Devouche, 2011).

Like ID speech, Infant Directed song (ID song), e.g., lullabies or play songs, has a distinctive style which involves high pitch, slow tempo, and other indices of heightened expressiveness or emotionality (Trainor et al., 1997; Trehub et al., 1997). Infants are more attentive to ID singing than to non-ID singing as newborns (Masataka, 1999) and at 6 months of age (Trainor, 1996). Though 5–6-month-old infants are highly attentive to maternal singing and talking they are considerably more engaged when listening to singing than to speech (Nakata and Trehub, 2004).

In addition to speech and song, music also plays a central role in eliciting infant communication. In particular, the presence of a regular beat in music facilitates emotional exchanges between the mother and infant, similar to the way it promotes coordinated movements and feelings between a group of musicians and a group of listeners (Benzon, 2002; Trehub, 2003). Interestingly, the effect of music persists even in the infants of deaf parents (Masataka, 1999). Brown (2003) portrays music as “the ideal synchronization device,” which may have evolved “to coordinate action and promote cohesion at the group level” (p. 16). In summary, as early as birth, rhythmical components of speech, song and music play important roles in enhancing infants’ arousal and their expression of vocal and body communicative interactions. However, an important question is whether neonates can engage in veridical sensorimotor synchronization, i.e., perceive an external rhythm and modify the tempo of their own spontaneous rhythms in synchrony with the external rhythm.

## SENSORIMOTOR SYNCHRONIZATION IN NEONATES

As mentioned at the beginning of this review, SMS is crucial to social interaction because it is an efficient way to communicate that the signals from another person’s behavior have been received. Adults and older children are able to adapt their spontaneous rhythm (Spontaneous Motor Tempo or SMT) to an external rhythm and their best SMS occurs when the external rhythm is around their own SMT. However, as reported previously, young children find it difficult to do the same; the intervals within which synchronization is possible are much narrower than the ones applicable to adults and they must be close to the natural tempo of the child’s movements. However, the limitations of SMS in young children do not imply that infants (even newborns) are unable to synchronize their primitive movements with an external rhythm or, at minimum, increase the frequency of their rhythms in response to external rhythms. As newborns can discriminate and produce rhythms, can they adapt these rhythmic behaviors to rhythmical stimulation? A positive response to this question would suggest that newborns can use their own rhythms to communicate with others, even if in a primitive form. To investigate this hypothesis, it is essential to use appropriate contexts in which newborns can produce rhythms and modify them according to perceived rhythms. Three newborn rhythmical activities have been tested for their potential adaptation to an external rhythm: non-nutritive sucking, stepping, and crying.

### NON-NUTRITIVE SUCKING IN RESPONSE TO AN AUDITORY RHYTHM

As described earlier, spontaneous non-nutritive sucking is a regular rhythmical activity performed by all newborns at birth at a spontaneous sucking period of approximately 450 ms (Bobin-Bègue et al., 2006). To test the early capacity of infants to adjust this sucking activity to an external rhythm, Bobin-Bègue et al. (2006) registered the sucking activity of 48 newborns and 18 2-month-olds with a sterilized pacifier connected to a pressure transducer and linked to a computer. First, the spontaneous motor tempo (SMT) was registered for each newborn during 110 sucks and then the SMT of each newborn was determined. In phase two of the experiment, sucking tempo was again registered in two conditions, (1) during 110 sucks while the infant heard an external auditory stimulus with a tempo that was identical to his/her SMT during the first phase (to habituate the infant to beats that were 65 db, 440 Hz, and 100 ms in length), then (2) during 110 sucks while the infant listened to a tempo that was accelerated or decelerated by 15% in comparison to each infant’s SMT. Finally, the newborn’s spontaneous motor tempo was recorded again during 110 sucks without any external stimulation. The goal of the study was to analyze the synchronization of the sucks with the auditory rhythm. A suck was classified as synchronized if it occurred during or before the auditory stimulation (within 15% of the Inter Stimulus Interval, ISI).

The results of the above-mentioned study showed that the 2-month-olds and the newborns were able to accelerate their sucking rhythm in the 15% accelerated auditory stimulation condition. However, only the 2-month-olds were able to decelerate their sucking rhythm when the auditory stimulation became 15% slower than their own rhythm. This result is not surprising given that newborns and 2-month-olds are able to decrease the pause duration between bursts of sucks (accelerating the sucking frequency), but newborns are not able to increase this pause (decelerating the sucking frequency) as reported by Pouthas et al. (1996). Regardless of age, infants who have to accelerate their sucking rhythm are more synchronized than infants who have to decelerate it. Finally, the percentage of synchronized sucks is significantly higher for 2-month-olds than newborns independent of the task of acceleration or deceleration. These results highlight that newborns and 2-month-olds are able to synchronize their sucking activity to an external rhythm even if it is within a narrow interval. While such sucking activity is not directly perceptible by an external observer, it may very well be perceived by the mother during her infant’s nutritive feeding or during non-sucking periods in the feeding, contributing to a very early interaction between the infant and the mother, especially if the mother uses ID speech or song when she feeds her infant.

### STEPPING IN RESPONSE TO AN AUDIO–VISUAL RHYTHM

We have already reported that newborns are able to make alternating rhythmic stepping movements when they are supported in the upright position with the soles of their feet touching a rigid surface. Many studies have shown that the frequency of newborn stepping is regulated by multiple internal and external factors, with the infant’s level of arousal playing a central role. For example, newborns step more when they are crying. Despite considerable attention to the factors that can modify the stepping pattern, nothing is known about the newborn’s capacity to adapt their stepping to an external tempo. To investigate this question, Provasi et al. (in preparation and partially reported in Provasi and Barbu-Roth, 2009) recently recorded the stepping activity of 40 3-day-old newborns (27 of whom stepped in each of the experimental conditions) in response to a bimodal audio–visual stimulus presented either continuously (without rhythm) or at three different temporal frequencies (with rhythm). The primary goal was to determine whether the rhythmic conditions elicited more stepping than the no rhythmic condition and whether one particular rhythmic condition would elicit more bursts of rhythmic stepping. A secondary goal was to determine whether steps would be synchronized with the external rhythm even though stepping, in contrast to sucking, is highly variable at this age (Barbu-Roth et al., 2009, 2013). Air stepping was chosen for the experimental conditions to determine whether stepping could be induced or facilitated by the audio–visual rhythmic stimuli alone, i.e., in the absence of tactile stimulation.

Significantly fewer steps were taken in the non-rhythmic condition than in the slowest rhythmic condition in Provasi et al.’s study. Consequently, rhythmical stimulation appeared to enhance motor production. To gain further insight into whether the conditions influenced stepping coordination, the number of alternating steps (the most rhythmical type of stepping movements) was determined using criteria developed by Groenen et al. (2010). Although only seven newborns exhibited at least three alternating steps per rhythmical condition, analyses indicated that significantly more alternating steps were taken when the audio–visual rhythm was slower (ISI = 1700 ms) than when it was faster (ISI = 1400 ms). Thus rhythmical stimulation appeared to enhance rhythmical production when the audio–visual rhythm was slow.

### CRYING IN RESPONSE TO AN AUDIO–VISUAL RHYTHM

Crying is the third example of a rhythmical behavior in newborns. The crying responses of 40 newborns were recorded during audio–visual rhythmical stimulation by Provasi and Barbu-Roth (2009) with the same experimental procedure as reported above. 33 neonates cried in a least one condition and 18 infants cried in the three rhythmic conditions. The analyses were done on these 18 neonates. Significantly fewer cries were detected in the non-rhythmic condition than in each of the rhythmic conditions. Thus rhythmical stimulation appears to enhance crying production in the same way it enhances stepping production. To determine the synchrony between the cries and the audio–visual stimulus, the circular statistics recommended by Patel et al. (2009) and Kirschner and Tomasello (2009) were used. Significantly more synchronized cries were observed for the slowest audio–visual stimulation period than for the two other audio–visual stimulation periods. This period (1700 ms) is close to the natural crying tempo (around 1500 ms). As most studies of the natural crying tempo have recorded crying production before feeding (Wolff, 1969; Brennan and Kirkland, 1979, 1982; Zeskind et al., 1992; Lin and Green, 2007), and in the current study the newborns were recorded after feeding, it is possible that the natural crying tempo after feeding might be slower than it is before feeding, i.e., around 1700 ms. If this is the case, this result would suggest that newborns are able to adapt their spontaneous crying tempo when the external tempo is close to their spontaneous crying tempo. In any case, newborns seem able to adapt their crying tempo to an external rhythmic stimulation.

## CONCLUDING COMMENTS

Isochronous synchronized behaviors may be viewed as the simplest building blocks for forming social bonds (Trevarthen, 1977, 1979, 1993; Trevarthen and Aitken, 2001; Merker et al., 2009; Wiltermuth and Heath, 2009; Phillips-Silver et al., 2010; Valdesolo et al., 2010). Moreover, as the ability to synchronize motor behavior with an external auditory beat is restricted to vocal learning species (Patel, 2006; Patel et al., 2009; Schachner et al., 2009), sensorimotor synchronization appears to play a crucial role in social interaction. This literature review has highlighted an important point central to our understanding of sensorimotor synchronization; rhythm is omnipresent in communication: to perceive and produce any communication signal we need rhythmical signals. As for adults, surrounding rhythms are part of the daily life of fetuses and newborns. These stimulations principally arise from the activities of the mother during pregnancy and the mother or caregiver after birth who, in close contact with the neonate, produces rhythmic patterns during breathing, cardiac activity, walking, speaking, singing, and moving their limbs or face in different cadences. These activities expose the infant to multiple patterns of repetitive vestibular, tactile, somatosensory, auditory, and visual stimulation. Many studies have revealed that fetuses and newborns detect these patterns and differentiate their variable rhythms. This is particularly true for auditory rhythms, which are detected very early and provide the basis for future speech and music production. However, neonates are not only surrounded by auditory rhythms but also by multiple VTS rhythms. These are experienced on a regular basis because the neonate is often transported and touched as this early age. Not surprisingly, neonates can not only recognize these different VTS rhythms, but also change their behavior in response to these rhythms, with the most commonly known effect being the calming response produced by cadenced oscillations (e.g., rocking and swaying).

Might VTS rhythms have a role in the future production of movements similar to the way in which auditory rhythms influence speech production? This question has never been explored and opens new directions for future research. Interestingly, VTS rhythms have been reported to influence basic infant movement patterns; for example, VTS stimulation increases breathing frequency in preterm infants, who adapt the rhythm of their breathing to the velocity of stimulation (Sammon and Darnal, 1994). If such an effect is obtained with an unfamiliar VTS stimulation, we might hypothesize that familiar VTS stimulations, such as those that result from a pregnant women walking, should elicit an even stronger effect on rhythm perception and production. In any case, future research is needed to investigate the role of VTS rhythms on rhythm perception, production, and synchronization, especially with regard to preterm infants who are deprived of such stimuli early on in their development.

This literature review shows that not only are fetuses and newborns immersed in rhythms and detect them very early, but that fetuses and newborns are able to produce many different rhythms. However, the occurrence and temporal frequency of this production varies considerably, depending on the activity observed. Activities like breathing and producing cardiac pulsations are necessarily regular and continuous as their function is to maintain life. In contrast, sucking, stepping, and crying have episodic functions and they emerge in irregular bursts. Consequently, cardiac pulsations and breathing rhythms are likely more responsive to external rhythms and may change quantitatively (frequency increase or decrease) or qualitatively (SMS). Actually, many studies have shown changes in the temporal frequency of HR with external rhythmical stimulations in fetuses and newborns. Moreover, the capacity of preterms to adapt their breathing rhythm to the velocity of a linear displacement of their body suggests some kind of primitive SMS. If this result is confirmed, the capacity could very well underlie a primitive form of communication as a change in neonatal breathing could be perceived by another person in close proximity to the infant.

With sucking, stepping, and crying, the situation is different. All these behaviors are produced spontaneously and irregularly and consequently they are difficult to use to determine whether neonates have the capacity to perform SMS. However, all can be elicited on a more regular basis if the neonate is placed in an appropriate context. This is what is observed with non-nutritive sucking, where a pacifier is used to stimulate the emergence of more regular bursts of sucking. In this case, it is possible to measure the Spontaneous Motor Tempo and see whether the tempo can be adapted to an external tempo. This technique allowed Bobin-Bègue and Provasi to determine for the first time that newborns can synchronize with an external tempo if the tempo is faster than 15% of their Spontaneous Motor Tempo. At this age, infants are not able to synchronize with a tempo slower than their Spontaneous Motor Tempo. In contrast, 2-month-old infants are able to accelerate their sucking tempo when the external rhythmical stimulation becomes faster and decelerate it when the external rhythmical stimulation slows down. However, it is an open question whether these changes in sucking tempo can be perceived by an external observer, especially when the SMS is performed within such a narrow range of tempi.

In contrast to sucking, stepping, and crying can easily be perceived by a caregiver, with crying being perceptible by someone located far away and unable to see the infant. As a result, these rhythmic activities are excellent candidates for communication. But how could a neonate indicate to another person, who is producing a specific rhythm, that this external rhythmical signal has been perceived? In this review, we have highlighted three ways for the infant to provide such information, with each solution being progressively more elaborated. First, the infant could simply increase the frequency of stepping or crying. Second, the infant could increase the number of rhythmic bursts of stepping or crying. Finally, the infant could perform a veridical SMS by mapping the occurrence of stepping or crying to the external tempo. The first strategy has been observed in both studies performed on crying and stepping: neonates increase their number of steps or cries in response to the slowest external tempo. Moreover, consistent with the second strategy, newborns produce more coordinated stepping (alternated steps) and more bursts of crying in the slowest rhythmic condition than in non-rhythmical condition. That was not the case for the two other rhythmical conditions. Finally, in the crying study, there was evidence that the newborns could not only increase their crying bursts, but also perform a primitive form of SMS in response to the specific tempo to which they were increasing their number of cries. This result is, to our knowledge, the first indication that neonates are not only able to perform SMS but are potentially able to communicate distally with others via SMS.

In summary, it appears that newborns are able to produce more coordinated and more synchronized motor behaviors in the presence of a specific rhythmical stimulus. Although these behaviors do not come close to the definition of sensorimotor synchronization put forward by Repp (2005), some primitive form of SMS appears to exist at birth. This conclusion brings us back to the original question posed at the beginning of this review: how can we improve the contexts in which we test neonatal SMS in order to facilitate such behavior and better investigate its characteristics? One possibility is to increase the regularity of the occurrence and the tempo of the rhythmic activity measured in the neonate. This is what a pacifier does to non-nutritive sucking; it increases the number of bursts and the spontaneous motor tempo. While it seems inconceivable to do the same with crying, some motivational factors might elicit more regular stepping, with the restriction, of course, that these factors do not contain any rhythmical information.

In conclusion, fetuses and newborns are able to produce many rhythmic activities, most of them generated automatically at a subcortical level, but modifiable by internal and external stimulation. The primary question is whether *the rhythm* of these activities is modifiable in response to an externally presented rhythm, indexed either by a change in the frequency of the rhythmic activity in response to a specific external tempo or by a veridical SMS with this tempo. Studies have rarely addressed this question in the fetus and few studies have been conducted with newborns. Preliminary results suggest that newborns are capable of a primitive form of SMS. However, future research, with appropriate contexts to facilitate SMS is needed to further explore this fascinating phenomenon. Understanding the early capacities of the fetus and newborns to synchronize their rhythms with those in the environment is key to understanding the origins of communication. One important factor to keep in mind is the contexts in which SMS might be facilitated. If, as researchers, we succeed in finding these contexts, then we will be able to investigate how SMS might be used by infants at risk of developing communication disorders to communicate.

## Conflict of Interest Statement

The authors declare that the research was conducted in the absence of any commercial or financial relationships that could be construed as a potential conflict of interest.
